# 非小细胞肺癌小活检标本质量与*EGFR*基因突变检出率关系分析

**DOI:** 10.3779/j.issn.1009-3419.2021.102.16

**Published:** 2021-05-20

**Authors:** 玉勤 李, 莹莹 顾, 桔红 姜

**Affiliations:** 510120 广州，广州医科大学附属第一医院，广州呼吸健康研究院呼吸疾病国家重点实验室/国家呼吸系统疾病临床医学研究中心 The State Key Laboratory of Respiratory Disease, Guangzhou Institute of Respiratory Health, National Clinical Research Center for Respiratory Disease, The First Affiliated Hospital, Guangzhou Medical University, Guangzhou 510120, China

**Keywords:** 肺肿瘤, 表皮生长因子受体突变, 肿瘤细胞数量, 肿瘤细胞比例, Lung neoplasms, Epidermal growth factor receptor mutation, Number of tumor cells, Proportion of tumor cells

## Abstract

**背景与目的:**

表皮生长因子受体（epidermal growth factor receptor, *EGFR*）是非小细胞肺癌（non-small cell lung cancer, NSCLC）患者中突变率最高的基因，准确检测出其突变类型有助于指导患者接受靶向药物治疗从而延长患者生存期。为了得到准确的检查结果，基因检测平台对标本质量有一定要求。已有文献报道标本的肿瘤细胞数量及其比例等会影响*EGFR*基因突变检出率，本研究旨在分析NSCLC小活检标本质量与突变扩增系统（amplification refractory mutation system, ARMS）法检测*EGFR*基因突变检出率的关系。

**方法:**

收集299例小活检肺腺癌病例的临床特征、在显微镜下评估切片的肿瘤细胞数量及比例、标本提取的DNA浓度、*EGFR*基因突变检测结果，分析标本质量与*EGFR*基因突变检出率的关系。

**结果:**

肿瘤细胞数≤500组与 > 500组*EGFR*基因突变阳性率分别为40.7%（11/27）、43.8%（119/272），两者间无统计学差异（*P*=0.764）。DNA浓度≤20.4 ng/μL组及 > 20.4 ng/μL组的基因突变阳性率分别为42.7%（64/150）、44.3%（66/149），两者间无统计学差异（*P*=0.776）。肿瘤细胞比例≤30%及 > 30%组的阳性率分别为29.4%（20/68）、47.6%（110/231），两者间存在统计学差异（*P*=0.008）。多因素*Logistic*分析显示男性、甲状腺转录因子1（thyroid transcription factor-1, TTF-1）阴性、有吸烟史及肿瘤细胞比例 < 30%是影响*EGFR*基因突变低检出率的主要因素。

**结论:**

在达到检测的最低要求后，肿瘤细胞比例仍可影响*EGFR*基因突变检出率。有必要在基因检测切片后再次评估最后一张切片的肿瘤细胞比例，对于肿瘤细胞比例过低的标本，建议通过显微切割等方法富集肿瘤细胞，提高其肿瘤细胞比例，得出更准确的检测结果。对于无法进行肿瘤细胞富集的标本，可行循环肿瘤DNA（circulating tumor DNA, ctDNA）检测作为补充，若结果仍为阴性才需考虑再次活检获取足够的肿瘤标本进行分子检测。

## Abstract (en)

**Background and objective:**

Epidermal growth factor receptor (*EGFR*) is the gene with the highest mutation rate in non-small cell lung cancer (NSCLC) patients, and the accurate evaluation of its mutational status can facilitate patients receiving targeted drug therapy and thereby prolong patients' survival. The gene testing platform has adequacy requirements for the specimen quality in order to obtain accurate examination results. It has been reported that the number and proportion of tumor cells in samples will affect the detection rate of *EGFR* gene mutation. The present study aims to analyze the relationship between the quality of small biopsy specimens of NSCLC and the mutation rate of *EGFR* gene with amplification refractory mutation system (ARSM) test.

**Methods:**

After collecting the clinical characteristics of 299 cases small biopsy of lung adenocarcinoma, DNA concentration of the specimens and the mutational status of *EGFR* gene, the number and proportion of tumor cells in HE stained sections evaluated using light microscopy, the relationship between specimen quality and the mutation rate of *EGFR* gene were analyzed.

**Results:**

The mutation rates of *EGFR* for the groups with tumor cell number ≤500 and > 500 were 40.7% (11/27) and 43.8% (119/272) respectively, without significant difference (*P*=0.764). The mutation rates for the groups with DNA concentration ≤20.4 ng/μL and > 20.4 ng/μL were 42.7% (64/150) and 44.3% (66/149) respectively, without significant difference (*P*=0.776). The mutation rates for the groups with tumor cells proportion ≤30% and > 30% were 29.4% (20/68) and 47.6% (110/231) respectively, demonstrating significant difference (*P*=0.008). Multivariate *Logistic* analysis showed that male, thyroid transcription factor-1 (TTF-1) negative, smoking history and tumor cell proportion less than 30% were main factors that contributes to the low detection rate of *EGFR* gene mutation.

**Conclusion:**

After meeting the minimum requirements for detection, the *EGFR* mutation rate is affected by the proportion of tumor cells in the sample. Therefore, it is necessary to re-evaluate the tumor cell proportion in the last section after the genetic test section. For samples with lower tumor cell proportion, enriching tumor cells through microdissection and other methods is recommended for a more accurate detection result. For specimens that cannot be enriched with tumor cells, circulating tumor DNA (ctDNA) test can be performed as a supplement. If the result is still negative, another biopsy should be considered to obtain enough tumor specimens for molecular testing.

肺癌作为发病率和死亡率最高的恶性肿瘤，是引起全世界癌症相关死亡的主要原因^[1]^。非小细胞肺癌（non-small cell lung cancer, NSCLC）约占所有肺癌病例的85%，且预后情况不佳^[2]^。约60%的NSCLC患者因诊断时病情已处于晚期或伴有转移无法进行手术^[3]^，只能通过小活检组织或细胞学标本进行诊断。携带不同表皮生长因子受体（epidermal growth factor receptor, EGFR）酪氨酸激酶结构域的一些特异性体细胞突变能预测NSCLC患者对各种酪氨酸激酶抑制剂（tyrosine kinase inhibitors, TKIs）的反应^[4-6]^，选择TKI药物治疗前需要检测*EGFR*基因突变状态。因此，现在肺癌的病理评估既需要通过组织形态学和免疫组化对肿瘤进行准确的分型，也需要通过肿瘤的分子分析确定*EGFR*基因突变状态以选择接受合适的TKIs治疗^[7, 8]^。小活检标本肿瘤细胞数量及比例均可能对检测结果产生影响，假阴性结果主要因为活检样本中肿瘤细胞的数量或比例不足^[9]^。为了得出准确的检测结果，在检测前对标本的质量进行评估是非常有必要的。本研究拟分析小活检标本中肿瘤细胞比例、肿瘤细胞数量以及DNA浓度3个标本质量指标对*EGFR*基因突变检出率的影响，同时探讨患者性别、年龄等临床特征与*EGFR*基因突变检出率的关系，旨在优化临床检测方案，进一步提高*EGFR*基因突变检出率，延长患者的长期生存率。

## 1 材料与方法

### 1.1 临床病例收集

本研究收集了2017年4月-2020年7月期间广州医科大学附属第一医院初次病理诊断为肺腺癌并于本院进行了*EGFR*基因21种突变检测的299个病例，均为小活检标本，取材方法包括经支气管镜肺活检术（transbronchial lung biopsy, TBLB）取材、经皮肺穿刺取材以及经超声内镜引导下的经支气管针吸活检（endobronchial ultrasound-guided transbrochial needle aspiration, EBUS-TBNA）取材。石蜡组织标本处理由本院呼吸病理中心完成，DNA提取和扩增以及检测由本院转化实验室完成，采用厦门艾德生物医药科技股份有限公司*EGFR*基因21种突变检测试剂盒。于本院的临床病例系统及病理信息系统查询并收集每例患者的年龄、性别、肿瘤原发灶-淋巴结-转移（tumor-node-metastasis, TNM）分期、吸烟史、标本来源及其取材方法以及免疫组织化学结果等信息后，调取每例切片重新进行质量评估，并收集所有标本进行基因突变检测的DNA浓度及检测结果。

### 1.2 标本质量评估

我们对所有患者进行诊断性免疫组化连续切片最后一张石蜡切片的肿瘤细胞数和比例进行了重新评估。在光学显微镜下观察整张切片每一块小组织或将切片分为若干个区域，依次计数每小块组织或区域的肿瘤细胞及正常细胞数量，计算肿瘤细胞总的数量及比例作为标本质量评估的指标。

### 1.3 统计学方法

使用SPSS 25.0统计软件对数据进行分析，各组统计采用行乘列表卡方检验及*Fisher*确切概率法分析，采用单因素和多因素*Logistic*回归分析各因素对突变检出率的影响。*P* < 0.05为差异有统计学意义。

## 2 结果

### 2.1 临床特征

本研究299例患者中男性占64.2%（192/299），女性占35.8%（107/299）；年龄范围为27岁-95岁，平均年龄为（61.3±11.1）岁。肿瘤TNM分期Ⅱ期占1.0%（3/299），III期占32.1%（96/299），IV期占66.9%（200/299）。有吸烟史占47.5%（142/299），无吸烟史占52.5%（157/299）。免疫组化甲状腺转录因子1（thyroid transcription factor-1, TTF-1）阳性的标本占89.3%（267/299），阴性占10.7%（32/299）。TBLB取材的标本占40.1%（120/299），经皮肺穿刺取材的标本占19.4%（58/299），EBUS-TBNA取材的标本占40.5%（121/299）。

### 2.2 *EGFR*基因突变检出情况

本次研究299例患者中共检出130例患者的142个*EGFR*基因突变位点。单一位点突变有118例，分别为：2例外显子18 G719X，61例外显子19缺失，2例外显子20插入突变、1例S768I，51例外显子21 L858R、1例L861Q。合并*EGFR*基因2个位点突变有12例，其中3例外显子18 G719A/719C合并外显子21 L861Q，1例外显子19缺失合并外显子21 L858R，2例外显子20 T790M合并外显子19缺失；6例T790M合并外显子21 L858R。

### 2.3 *EGFR*基因突变阳性率与临床特征的关系

男性的*EGFR*基因突变阳性率为32.3%（62/192），女性阳性率为63.6%（68/107），两者间存在统计学差异（*P* < 0.001）。年龄≤50岁组的阳性率为46.2%（18/39）， > 50岁组的阳性率为43.1%（112/260），两者间无统计学差异（*P*=0.718）。TNM分期Ⅱ期组的阳性率为0%（0/3），Ⅲ期组的阳性率为43.8%(42/96)，Ⅳ期组的阳性率44.0%（88/200），三者之间无统计学差异（*P*=0.251）。有吸烟史组的阳性率为26.1%（37/142），无吸烟史组的阳性率为59.2%（93/157），两者间存在统计学差异（*P* < 0.001）。免疫组化TTF-1阳性组基因突变阳性率为47.6%（127/267），阴性组阳性率为9.4%（3/32），两者间存在统计学差异（*P* < 0.001）。TBLB取材组的基因突变阳性率为48.3%（58/120），经皮肺穿刺取材组的阳性率为37.9%（22/58），EBUS-TBNA取材组的阳性率为41.3%（50/121），三者之间无统计学差异（*P*=0.349）（表 1）。

**表 1:** 299例的临床特征及其与*EGFR*基因突变率的关系 Clinical characteristics of the 299 cases and their relationship with *EGFR* gene mutation rate

Clinical characteristics	Number	Mutation	*P*
Gender			< 0.001
Male	192	62 (32.3%)	
Female	107	68 (63.6%)	
Age (yr)			0.718
≤50	39	18 (46.2%)	
> 50	260	112 (43.1%)	
TNM stage			0.251
Ⅱ	3	0	
Ⅲ	96	42 (43.8%)	
Ⅳ	200	88 (44.0%)	
Smoking history			< 0.001
Yes	142	37 (26.1%)	
No	157	93 (59.2%)	
TTF-1			< 0.001
Positive	267	127 (47.6%)	
Negative	32	3 (9.4%)	
Procedure			0.349
TBLB	120	58 (48.3%)	
PNLB	58	22 (37.9%)	
EBUS-TBNA	121	50 (41.3%)	
Total			
Mutation	130	130 (43.5%)	
Wild type	169	0	
EGFR: epidermal growth factor receptor; TNM: tumor-node-metastasis; TTF-1: thyroid transcription factor-1; TBLB: transbronchial lung biopsy; PNLB: percutaneous needle lung biopsy; ctDNA: circulating tumor DNA; ARMS: amplification refractory mutation system; NGS: next-generation sequencing; EBUS-TBNA: endobronchial ultrasound-guided transbronchial needle aspiration.										

### 2.4 *EGFR*基因突变检出率与标本质量的关系

我们经评估所有标本的苏木精-伊红染色法（hematoxylin-eosin staining, HE）切片，其中对应切片肿瘤细胞数 > 500占91.0%（272/299），≤500占9.0%（27/299），肿瘤细胞数≤500组与 > 500组的*EGFR*基因突变阳性率分别为40.7%（11/27）及43.8%（119/272），两者间无统计学差异（*P*=0.764）（表 2）。标本提取的DNA浓度范围：0.80 ng/μL-645.30 ng/μL，中位数为20.4 ng/μL，浓度≤20.4 ng/μL组及 > 20.4 ng/μL组的*EGFR*基因突变阳性率分别为42.7%（64/150）、44.3%（66/149），两者间无统计学差异（*P*=0.776）（表 3）。299例标本对应切片的肿瘤细胞比例范围为10%-90%，肿瘤细胞比例≤30%占22.7%（68/299）， > 30%占77.3%（231/299），阳性率分别为29.4%（20/68）、47.6%（110/231），两者间存在统计学差异（*P*=0.008）（表 4）。299例标本的外显子20 T790M突变阳性率为2.7%（8/299），肿瘤细胞比例≤30%组的阳性率为1.5%（1/68）， > 30%组的阳性率为3.0%（7/231），两者间无统计学差异（*P*=0.688）。

**表 2:** 癌细胞数量与*EGFR*基因突变率的关系 Relationship between the number of tumor cells and *EGFR* gene mutation rate

Number	Wildtype	Mutation	Mutation rate	*χ*^2^	*P*
≤500	16	11	40.7%	0.091	0.764
> 500	153	119	43.8%		

**表 3:** DNA浓度与*EGFR*基因突变率的关系 Relationship between DNA concentration and *EGFR* gene mutation rate

DNA concentration	Wildtype	Mutation	Mutation rate	*χ*^2^	*P*
≤20.4 ng/*μ*L	86	64	42.7%	0.081	0.776
> 20.4 ng/*μ*L	83	66	44.3%		

**表 4:** 癌细胞比例与*EGFR*基因突变率的关系 Relationship between the tumor cell propotion and mutation rate of *EGFR* gene

Proportion	Wildtype	Mutation	Mutation rate	*χ*^2^	*P*
≤30%	48	20	29.4%	7.087	0.008
> 30%	121	110	47.6%		

**表 5:** 多因素*Logistic*回归分析各因素对*EGFR*基因突变检出率的影响 Analyzing the influence of various factors on *EGFR* gene mutation detection rate with multivariate *Logistic* regression

Factors	Univariate *Logistic* analysis			Multivariate *Logistic* analysis	
	OR (95%CI)	*P*		OR (95%CI)	*P*
Male	3.655 (2.225-6.006)	< 0.001		2.323 (1.266-4.263)	0.006
Smoking history	4.125 (2.522-6.742)	< 0.001		3.252 (1.831-5.777)	< 0.001
TTF-1 negative	10.462 (2.413-45.351)	0.002		7.914 (1.789-35.015)	0.006
Tumor cell number	1.131 (0.506-2.528)	0.764			
DNA concentration	1.069 (0.676-1.688)	0.776			
Tumor cell proportion≤30%	2.119 (1.182-3.797)	0.001		2.134 (1.045-4.358)	0.038

### 2.5 单因素及多因素*Logistic*回归分析

单因素*Logistic*分析表明，男性（OR=3.655, 95%CI: 2.225-6.006, *P* < 0.001）、有吸烟史（OR=4.125, 95%CI: 2.522-6.742, *P* < 0.001）、TTF-1阴性（OR=10.462, 95%CI: 2.413-45.351, *P*=0.002）及肿瘤细胞比例≤30%（OR=2.119, 95%CI: 1.182-3.797, *P*=0.001）均与低突变检出率有关。多因素*Logistic*分析表明，男性（OR=2.323, 95%CI: 1.266-4.263, *P* < 0.001）、有吸烟史（OR=3.252, 95%CI: 1.831-5.777, *P* < 0.001）、TTF-1阴性（OR=7.914, 95%CI: 1.789-35.015, *P*=0.006）及肿瘤细胞比例 < 30%（OR=2.134, 95%CI: 1.045-4.358, *P*=0.038）是与低突变检出率有关的主要因素。

## 3 讨论

随着分子医学和靶向药物研究的发展，肺癌的治疗已经进入到个体化治疗的阶段。伴有*EGFR*基因突变的不可手术晚期非小细胞肺癌患者接受TKI靶向药物治疗，其疗效显著优于含铂化疗，能较大程度改善患者的生活质量。准确地检测出*EGFR*基因突变型是选择合适靶向药物的重要前提，而标本的质量显著影响结果的准确性，标本质量不佳是造成假阴性结果的主要原因，因此在检测前对标本进行质量评估能有效避免这种现象的出现，从而获得准确的检测结果，使患者最大程度受益。本次分析设定了3个标本质量评估指标，分别为标本提取的基因组DNA浓度、对应切片的肿瘤细胞数量以及肿瘤细胞比例。我们的分析结果显示DNA浓度≤20.4 ng/μL组及 > 20.4 ng/μL组的阳性率之间无统计学差异（42.7% *vs* 44.3%）。有研究^[10]^数据显示基因突变阳性组和阴性组的DNA浓度平均值及95%CI之间没有统计学差异（*P* > 0.05）。我们认为样本提取的DNA浓度在高于2 ng/μL达到合格水平后，DNA浓度的高低不再影响*EGFR*基因突变阳性率。在我们的分析中，在肿瘤细胞数 > 200的前提下，肿瘤细胞数≤500组（40.7%）与 > 500组（43.8%）的阳性率没有统计学差异，说明肿瘤细胞数 > 200之后，肿瘤细胞总数与阳性率无明显相关性。有研究^[11]^的实验数据显示突变阳性组和阴性组的肿瘤细胞数中位数之间没有统计学差异（*P*=0.172）。因此，只要不低于一个特定的下限值（与检测方法有关），肿瘤细胞数量并不影响阳性率，本研究的结论与此一致。文献报道及我们的结果均表明石蜡切片的肿瘤细胞数量及其提取的DNA浓度都不是影响阳性率的主要因素，但我们发现肿瘤细胞比例≤30%组和 > 30%组检出率分别为29.4%、47.6%，肿瘤细胞比例与阳性率存在明显相关性。

灵敏度不同的检测方法要求的肿瘤细胞比例的最小检测阈值是不相同的。直接测序法要求组织切片肿瘤细胞比例需达到30%-50%^[12]^；低温变性共扩增法（coamplification at lower denaturation temperature-polymerase chain reaction, COLD-PCR）要求的检测阈值是30%^[13]^，而灵敏度更高的是突变扩增系统（amplification refractory mutation system, ARMS），ARMS法作为一种基于突变等位基因优先扩增的高灵敏度检测方法，其原理是特异性引物与突变DNA模板互补结合扩增，而野生型模板则不能与引物成功配对。但是当标本肿瘤细胞比例过低时，大量野生型DNA的存在可能会影响突变DNA模板与特异性引物的结合导致扩增失败。该法要求肿瘤细胞绝对数量 > 200个，肿瘤细胞比例 > 10%就能成功检测出突变，这种高灵敏度的方法已经广泛应用于临床对肺癌患者的*EGFR*基因突变检测。本次分析采用的即为这种检测方法，我们病例标本检测前评估的肿瘤细胞数和比例都达到检测平台的要求，但是有一部分肿瘤细胞比例在10%-30%之间的病例，其*EGFR*突变的检出率较低。我们认为造成这种现象有两个原因：①评估人员的主观性，根据文献^[14, 15]^报道，发现病理医生视觉评估的肿瘤细胞比例会比使用扫描镜系统计算的比例高10%-20%；②连续切片会导致肿瘤组织的损耗，用于评估肿瘤细胞数量和比例的切片来自于切取分子检测的切片前的切片，后续的连续切片会导致肿瘤组织的损耗，因此实际用于*EGFR*基因突变检测的切片肿瘤细胞比例可能会比检测前镜下评估的切片比例低。这可能是造成肿瘤细胞比例镜下评估值在10%-30%的标本其突变检出率较低的另一个原因。为了提高*EGFR*基因突变检出率，我们最好对分子检测切片前后的HE切片均进行标本质量的评估，这样才能真实反映分子检测标本的肿瘤细胞数和比例。另外，当标本肿瘤细胞比例低时，有必要通过显微切割等方法富集肿瘤细胞，提高肿瘤细胞比例以达到检测阈值^[16]^。

近年来，下一代测序（next-generation sequencing, NGS）已广泛用于临床分子检测。与传统方法相比，这种新技术的最大优点是能够在一次检测中检测到多种基因改变及伴随突变，且能定量检测突变等位基因频率^[17]^。NGS分析灵敏度为肿瘤细胞比例的5%-10%^[18]^，一般建议将送检阈值定为灵敏度的2倍以上^[19]^，部分研究采用的送检阈值为10%^[20]^，而部分研究为20%^[18, 21, 22]^，其中存在10%的差异。鉴于我们发现ARMS法肿瘤细胞比例范围在10%-30%的标本阳性率低于肿瘤细胞比例 > 30%组，我们推测使用NGS进行检测的标本也有可能也会存在类似情况，肿瘤细胞比例范围在临界状态（10%-20%）的样本可能也会存在部分假阴性情况。目前只有一项研究对比了肺腺癌肿瘤细胞比例10%-20%的样本与 > 20%的标本使用NGS方法检测突变的阳性率，结果发现*EGFR*和鼠类肉瘤病毒癌基因（kirsten ratsarcoma viral oncogene, KRAS）基因的检出率在两者中无明显差异，但肿瘤细胞比例10%-20%组的*HER2*/*BRAF*/*PIK3CA*和获得性*EGFR* T790M突变的检出率低于肿瘤细胞比例 > 20%组^[23]^。我们的研究也发现肿瘤细胞比例≤30%组T790M突变阳性率低于肿瘤细胞比例 > 30%组，但是可能肿瘤细胞比例≤30%组样本量不够大，无统计学差异（*P*=0.688）。关于肿瘤细胞比例范围在10%-20%这类临界状态的标本使用NGS方法得出的阴性结果是否可信、*EGFR*及其他驱动基因检出率是否也会受肿瘤细胞比例低的影响、是否需要将送检阈值提高等问题值得进一步研究。而对于部分石蜡组织肿瘤细胞比例低、无法进行富集而再次活检取材风险大的患者，血液中的循环肿瘤DNA（circulating tumor DNA, ctDNA）是一种常用的突变检测肿瘤DNA替代来源。目前检测ctDNA的方法并未标准化，几项研究得出的ctDNA检测敏感性范围在66%-78%^[24-27]^，与石蜡组织的敏感性相比较低。但液体活检取材无侵入性创伤且操作简便，可与石蜡组织相结合诊断。当检测标本石蜡组织肿瘤细胞比例低于30%怀疑结果为假阴性时，可补充行ctDNA检测，若检测结果为阳性可避免二次活检带来的风险，若结果仍为阴性才需考虑再次活检获取足够的肿瘤标本进行分子检测。

本研究结果表明，在达到检测的最低要求后，DNA浓度和肿瘤细胞的绝对数量并不影响*EGFR*基因突变检出率，但是低肿瘤细胞比例仍可影响*EGFR*基因突变检出率。因此，基因检测切片后，有必要再次评估最后一张切片以得出准确的送检标本肿瘤细胞比例。对于比例较低的标本有必要通过显微切割等方法富集肿瘤细胞，避免比例低导致假阴性结果。对于无法进行肿瘤细胞富集的标本，可行ctDNA检测作为补充，若结果仍为阴性才需考虑再次活检获取足够的肿瘤标本进行分子检测。

## References

[1] Bray F, Ferlay J, Soerjomataram I (2018). Global cancer statistics 2018: GLOBOCAN estimates of incidence and mortality worldwide for 36 cancers in 185 countries. CA Cancer J Clin.

[2] Torre LA, Bray F, Siegel RL (2015). Global cancer statistics, 2012. CA Cancer J Clin.

[3] De Biase D, Visani M, Malapelle U (2013). Next-generation sequencing of lung cancer EGFR exons 18-21 allows effective molecular diagnosis of small routine samples (cytology and biopsy). PLoS One.

[4] Kulesza P, Ramchandran K, Patel JD (2011). Emerging concepts in the pathology and molecular biology of advanced non-small cell lung cancer. Am J Clin Pathol.

[5] Travis WD, Brambilla E, Noguchi M (2011). International Association for the Study of Lung Cancer/American Thoracic Society/European Respiratory Society International Multidisciplinary Classification of Lung Adenocarcinoma. J Thorac Oncol.

[6] Pirker R, Herth FJ, Kerr KM (2010). Consensus for *EGFR* mutation testing in non-small cell lung cancer: results from a European workshop. J Thorac Oncol.

[7] Beau-Faller M, Degeorges A, Rolland E (2011). Cross-validation study for epidermal growth factor receptor and KRAS mutation detection in 74 blinded non-small cell lung carcinoma samples: a total of 5, 550 exons sequenced by 15 molecular French laboratories (evaluation of the *EGFR* mutation status for the administration of EGFR-TKIs in non-small cell lung carcinoma[ERMETIC] project--part 1). J Thorac Oncol.

[8] Pao W, Girard N (2011). New driver mutations in non-small-cell lung cancer. Lancet Oncol.

[9] Scarpino S, Pulcini F, Di Napoli A (2015). *EGFR* mutation testing in pulmonary adenocarcinoma: evaluation of tumor cell number and tumor percent in paraffin sections of 120 small biopsies. Lung Cancer.

[10] Tam IY, Leung EL, Tin VP (2009). Double *EGFR* mutants containing rare *EGFR* mutant types show reduced *in vitro* response to gefitinib compared with common activating missense mutations. Mol Cancer Ther.

[11] Yatabe Y, Kosaka T, Takahashi T (2005). *EGFR* mutation is specific for terminal respiratory unit type adenocarcinoma. Am J Surg Pathol.

[12] Smouse JH, Cibas ES, Janne PA (2009). *EGFR* mutations are detected comparably in cytologic and surgical pathology specimens of non-small cell lung cancer. Cancer.

[13] Santis G, Angell R, Nickless G (2011). Screening for EGFR and KRAS mutations in endobronchial ultrasound derived transbronchial needle aspirates in non-small cell lung cancer using COLD-PCR. PLoS One.

[14] Warth A, Penzel R, Brandt R (2012). Optimized algorithm for Sanger sequencing-based *EGFR* mutation analyses in NSCLC biopsies. Virchows Arch.

[15] Forest F, Stachowicz ML, Casteillo F (2017). EGFR, KRAS, BRAF and HER2 testing in metastatic lung adenocarcinoma: Value of testing on samples with poor specimen adequacy and analysis of discrepancies. Exp Mol Pathol.

[16] Aisner DL, Deshpande C, Baloch Z (2013). Evaluation of *EGFR* mutation status in cytology specimens: an institutional experience. Diagn Cytopathol.

[17] Haley L, Tseng LH, Zheng G (2015). Performance characteristics of next-generation sequencing in clinical mutation detection of colorectal cancers. Mod Pathol.

[18] Roy-Chowdhuri S, Goswami RS, Chen H (2015). Factors affecting the success of next-generation sequencing in cytology specimens. Cancer Cytopathol.

[19] Wong NA, Gonzalez D, Salto-Tellez M (2014). RAS testing of colorectal carcinoma-a guidance document from the Association of Clinical Pathologists Molecular Pathology and Diagnostics Group. J Clin Pathol.

[20] Baum JE, Zhang P, Hoda RS (2017). Accuracy of next-generation sequencing for the identification of clinically relevant variants in cytology smears in lung adenocarcinoma. Cancer Cytopathol.

[21] Hwang DH, Garcia EP, Ducar MD (2017). Next-generation sequencing of cytologic preparations: An analysis of quality metrics. Cancer Cytopathol.

[22] Stoy SP, Segal JP, Mueller J (2018). Feasibility of Endobronchial ultrasound-guided transbronchial needle aspiration cytology specimens for next generation sequencing in non-small-cell lung cancer. Clin Lung Cancer.

[23] Li WH, Qiu T, Ling Y (2018). Subjecting appropriate lung adenocarcinoma samples to next-generation sequencing-based molecular testing: challenges and possible solutions. Mol Oncol.

[24] Zhou C, Wu YL, Chen G (2011). Erlotinib versus chemotherapy as first-line treatment for patients with advanced *EGFR* mutation-positive non-small-cell lung cancer (OPTIMAL, CTONG-0802): a multicentre, open-label, randomised, phase 3 study. Lancet Oncol.

[25] Mok T, Wu YL, Lee JS (2015). Detection and dynamic changes of *EGFR* mutations from circulating tumor DNA as a predictor of survival outcomes in NSCLC patients treated with first-line intercalated erlotinib and chemotherapy. Clin Cancer Res.

[26] Karachaliou N, Mayo-de las CC, Queralt C (2015). Association of *EGFR* L858R mutation in circulating free DNA with survival in the EURTAC trial. JAMA Oncol.

[27] Douillard JY, Ostoros G, Cobo M (2014). Gefitinib treatment in *EGFR* mutated Caucasian NSCLC: circulating-free tumor DNA as a surrogate for determination of *EGFR* status. J Thorac Oncol.

